# Body Composition by Bioelectrical Impedance Analysis: Associations with Nutritional Status, Functional Limitations, and Chronic Diseases in Older Adults

**DOI:** 10.3390/nu18060969

**Published:** 2026-03-19

**Authors:** Anna Tomasiewicz, Beata Jankowska-Polańska, Sebastian Makuch, Jacek Polański, Wojciech Tański

**Affiliations:** 1 4th Military Clinical Hospital in Wrocław, 50-981 Wrocław, Poland; 2 Faculty of Medicine, Wrocław University of Science and Technology, 50-376 Wrocław, Poland; 3 Department of Clinical and Experimental Pathology, Wrocław Medical University, 50-368 Wrocław, Poland; s.makuch@umw.edu.pl; 4 Department of Internal and Occupational Diseases, Hypertension and Clinical Oncology, Wrocław Medical University, 50-556 Wrocław, Poland

**Keywords:** elderly, electrical bioimpedance, nutritional status, functional limitations, chronic diseases

## Abstract

**Background**: Changes in body composition, such as decreased muscle mass and increased adipose tissue, are significant in older adults, impacting health, functional capacity, and increasing the risk of metabolic diseases, functional decline, and frailty. Bioelectrical Impedance Analysis (BIA) is a non-invasive tool for assessing body composition, including fat-free mass (FFM), skeletal muscle mass (SMM), and fluid distribution (e.g., ECW/TBW ratio). Complementing BIA, the Mini Nutritional Assessment (MNA) serves as a validated tool for identifying malnutrition risk in the elderly. This study aimed to understand the correlation between BIA-derived parameters, MNA scores and clinical outcomes. **Methods**: This cross-sectional study involved 195 older adults (mean age 72.8 ± 5.4 years), divided into two groups based on body composition profiles determined by cluster analysis. Data collected included demographics, comprehensive BIA parameters (BMI, fat mass, FFM, SMM, ECW/TBW, phase angle), MNA scores, self-assessed health, chronic disease prevalence, frailty index (TFI), and functional limitations (EQ-5D). Statistical analyses included descriptive statistics, *t*-tests/ANOVA, chi-square tests, Pearson’s/Spearman’s correlations, point-biserial correlations, regression analyses, and ROC curve analysis to compare groups, explore variable relationships, and assess predictive abilities for malnutrition risk. **Results**: The first group had significantly higher BMI, AFM (AFM), FFM, and SMM, but a lower ECW/TBW ratio compared to Group 2 (*N* = 115), which was predominantly female and had higher frailty scores. MNA scores showed significant positive correlations with FFM (rho = 0.165, *p* = 0.021) and SMM (rho = 0.182, *p* = 0.011), and a negative correlation with ECW/TBW (rho = −0.188, *p* = 0.008). Higher adiposity (BMI, fat mass) correlated positively with arterial hypertension and obesity. Lower FFM and SMM were negatively correlated with gastroesophageal reflux disease. Skeletal muscle mass (AUC = 0.634, cut-off ≤ 17.3 kg) and ECW/TBW ratio (AUC = 0.626, cut-off ≥ 49.7%) showed modest discriminatory capacity to identify malnutrition risk. Individuals at risk of malnutrition reported greater functional limitations and lower self-assessed health. Numerous BIA parameters, including segmental muscle mass, total body water, phase angle, and impedance values, significantly correlated with MNA scores. **Conclusions**: The study highlights the importance of body composition analysis, particularly BIA, in correlation with MNA, for assessing nutritional status, functional limitations, and chronic disease associations in older adults. Integrating BIA and MNA into geriatric assessments provides a complementary profile of nutritional and functional vulnerability.

## 1. Introduction

Body composition is a critical determinant of health and well-being, particularly in older adults. Age-related changes in body composition, such as a progressive decline in skeletal muscle mass and strength (sarcopenia) and an increase in adipose tissue, can significantly affect overall health and quality of life [1]. These changes are often associated with an increased risk of adverse health outcomes, including functional decline, frailty, and mortality [2]. Hence, understanding the specific changes in these components, rather than relying solely on global metrics such as weight, is critical for detecting at-risk individuals.

Factors like decline in muscle mass, increase in fat mass, and alterations in body fluid distribution have all been associated with compromised mobility, reduced physical performance, and impaired overall well-being [2,3,4]. The relationship between body composition and nutritional status is well known and broadly researched. While anthropometric measures such as BMI, fat mass (FM), and fat-free mass (FFM) can be used to assess the risk of malnutrition and predict adverse health outcomes, more detailed assessment methods are needed. To address this need, Bioelectrical Impedance Analysis (BIA) has emerged as a useful non-invasive, portable, and relatively inexpensive tool for estimating various body composition parameters. It allows us to simultaneously assess FFM, skeletal muscle mass (SMM), FM, total body water (TBW), and the distribution of water between extracellular (ECW) and intracellular (ICW) compartments [5,6]. The ECW/TBW ratio, for instance, can provide insights into fluid balance and cellular integrity. When assessing nutritional status in a geriatric population, a higher ECW/TBW ratio reflects a shift in fluids from the intracellular to the extracellular compartment, signifying a decline in metabolically active tissue and acting as a non-invasive proxy for sarcopenia and frailty [4]. Concurrently, the Mini Nutritional Assessment (MNA) is a widely validated and extensively used screening tool designed specifically to identify older adults who are malnourished or at risk of malnutrition [7]. The MNA incorporates a range of questions covering dietary intake, weight loss, mobility, psychological stress, neuropsychological problems, and anthropometric measurements. It synthesizes all of the aforementioned factors and creates a composite score that categorizes individuals into well-nourished, at risk of malnutrition, or malnourished [7].

Given the clinical significance of both body composition and nutritional status in the elderly, understanding the correlation between objective BIA-derived measures and established nutritional screening tools like the MNA is of considerable importance. Establishing strong correlations between specific BIA parameters and MNA scores can enhance the utility of BIA in clinical practice. In this study, we aimed to explore how body composition, measured through bioelectrical impedance analysis (BIA), relates to nutritional status, functional limitations, and the presence of chronic diseases in an aging population.

## 2. Materials and Methods

### 2.1. Variables

This study employed a cross-sectional design to compare two distinct groups of older adults (*N* = 195) based on their body composition profiles. The inclusion criteria for the study were an age of 58 years or older and the ability to provide written informed consent. Individuals with implanted electrical devices (e.g., cardiac pacemaker), generalised edema, and acute or unstable chronic conditions that precluded the safe performance of BIA measurements were excluded. The groups were differentiated by key body composition parameters such as Body Mass Index (BMI), fat mass (FM), and fat-free mass (FFM). A comprehensive set of variables was assessed, including demographic characteristics (age, gender, education, marital status, housing status, net income), body composition parameters (BMI, FFM, relative fat mass, absolute fat mass, skeletal muscle mass), and nutritional status (Mini Nutritional Assessment score) and self-assessment of health (score on a 0–100 scale). Additionally the prevalence of selected chronic diseases, including arterial hypertension, coronary heart disease, heart failure, atrial fibrillation/arrhythmia, atherosclerosis, high cholesterol, obesity, diabetes type 1, diabetes type 2, thyroid diseases, asthma, COPD, and respiratory failure, was evaluated. The total number of chronic diseases was also recorded. Furthermore, frailty was assessed using Tilburg Frailty Indicator (TFI; total and social domain), functional limitations were evaluated using the EQ-5D questionnaire (mobility, self-care, usual activities, pain/discomfort, anxiety/depression; Total score 1–4), and quality of life was measured using a 0–100 scale. Most importantly a range of bioelectrical impedance analysis (BIA) results were calculated, including skeletal muscle mass in different body segments, total body water, extracellular water, extracellular water to total body water ratio (ECW/TBW), weight, resting energy expenditure, fat-free mass index (FFMI), bioelectric impedance vector analysis parameters, phase angle as well as bioimpedance resistance and reactance at various frequencies. Self-assessment of lifestyle was evaluated using a single-item question integrated into the sociodemographic survey: ‘Generally speaking, how do you rate your lifestyle in terms of health?’. Participants categorised their lifestyle on a 3-point scale (1 = unhealthy lifestyle, 2 = neither healthy nor unhealthy lifestyle, 3 = healthy lifestyle). Data were collected by the use of anthropometric measurements (for BMI calculation) and bioelectrical impedance analysis to assess body composition and fluid distribution.

### 2.2. BIA Measurement Protocol

Bioelectrical impedance analysis was performed using a medical-grade SECA MBCA 555 device (seca, Hamburg, Germany). To ensure measurement accuracy, participants were assessed in the morning following an overnight fast of at least 8 h. They were instructed to abstain from alcohol for 24 h and vigorous physical activity for 12 h prior to the assessment, and were asked to void their bladders within 30 min of the measurement

### 2.3. Statistical Analysis

Statistical analyses were conducted to compare the two groups and explore the relationships between the measured variables. Descriptive statistics, including frequencies, means, standard deviations, medians, and interquartile ranges, were used to summarize the characteristics of the study sample and the two groups. Comparative analyses between Group 1 and Group 2 were performed using *t*-tests or ANOVA for continuous variables and chi-square tests for categorical variables to identify statistically significant differences. Correlations between variables were assessed using Pearson’s or Spearman’s rank correlation coefficients, depending on the nature and distribution of the data. Point-biserial correlation coefficients were used to assess the relationship between continuous and dichotomous variables. Regression analyses (linear and logistic) and Receiver Operating Characteristic (ROC) curve analysis were employed to identify factors influencing nutritional status and body composition and to evaluate the predictive ability of body composition parameters for malnutrition risk. To identify natural body composition phenotypes, a k-means cluster analysis was performed using Euclidean distance, inputting BMI, absolute fat mass, and fat-free mass.

## 3. Results

### 3.1. Sample Characteristics and Group Comparisons

The study included 195 older adults (mean age 72.8 ± 5.4 years), divided into two groups based on body composition patterns determined via cluster analysis: Group 1 (*N* = 80) and Group 2 (*N* = 115) (Table 1). The two groups showed significant differences in several characteristics. Group 2 had a significantly higher proportion of females (95.7% vs. 58.7%, *p* < 0.001). Group 1 participants were more likely to be married (51.3% vs. 28.7%) and less likely to be widowed (28.7% vs. 45.2%) compared to Group 2 (*p* = 0.006). Consequently, individuals in Group 1 were less likely to live alone (41.3% vs. 66.1%) and more likely to live with a spouse (50.0% vs. 25.2%) than those in Group 2 (*p* = 0.001). It is important to note the demographic disparity between the groups: Group 2 was mostly female (95.7%) compared to Group 1 (58.7%).

**Figure 1:**
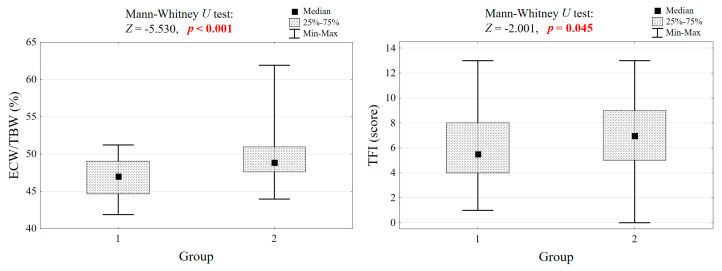
Comparison of extracellular water to total body water ratio (ECW/TBW) and frailty index (TFI) in 195 seniors across groups with different body mass compositions.

Significant differences in body composition were observed between the groups. Group 1 exhibited significantly higher mean BMI (30.4 ± 3.8 vs. 25.2 ± 3.3 kg/m^2^, *p* < 0.001), absolute fat mass (AFM) (34.0 ± 8.1 vs. 25.4 ± 6.1 kg, *p* < 0.001), fat-free mass (FFM) (51.2 ± 8.1 vs. 37.5 ± 4.8 kg, *p* < 0.001), and skeletal muscle mass (23.8 ± 4.6 vs. 15.8 ± 3.0 kg, *p* < 0.001) compared to Group 2. Despite these differences in the aforementioned parameters, relative fat mass (RFM) did not significantly differ between the groups (*p* = 0.948). Group 1 had a significantly lower extracellular water to total body water ratio (ECW/TBW) than Group 2 (46.9 ± 2.6% vs. 49.4 ± 2.7%, *p* < 0.001) (Figure 1).

No significant differences were found between the groups regarding age, education level, net income, MNA scores, nutritional status categories (proper nutrition vs. risk of malnutrition), or self-assessment of health. However, Group 2 showed significantly higher scores of the Tilburg Frailty Indicator (TFI) (6.6 ± 2.7 vs. 5.9 ± 2.9, *p* = 0.045) and the TFI social domain (2.0 ± 0.8 vs. 1.7 ± 0.9, *p* = 0.023). Regarding chronic diseases, obesity was significantly more prevalent in Group 1 (26.3% vs. 10.4%, *p* = 0.006), while gastroesophageal reflux disease was significantly more prevalent in Group 2 (33.0% vs. 18.8%, *p* = 0.033). The total number of chronic diseases did not differ significantly between the groups.

### 3.2. Comparison of Nutritional Status and Chronic Disease Prevalence Between Groups

The mean MNA score was 25.8 ± 2.5 in Group 1 and 25.2 ± 2.7 in Group 2, with no statistically significant difference (*p* = 0.081). Similarly, the distribution of nutritional status categories (proper nutrition vs. risk of malnutrition) did not differ significantly between the groups (*p* = 0.108). Self-assessment of health scores was also comparable (Group 1: 71.5 ± 15.2, Group 2: 72.1 ± 15.9, *p* = 0.767).

However, significant differences were observed in the prevalence of certain chronic diseases. Obesity was significantly more prevalent in Group 1 (26.3%) compared to Group 2 (10.4%, *p* = 0.006), which is consistent with the higher BMI in Group 1. Gastroesophageal reflux disease was markedly more frequent in Group 2 (33.0%) than in Group 1 (18.8%, *p* = 0.033). Although not statistically significant, there was a trend towards a higher prevalence of arterial hypertension in Group 1 (55.0%) compared to Group 2 (40.9%, *p* = 0.059). The prevalence of all other chronic diseases listed in Table 1 did not differ significantly between the two groups. The mean number of chronic diseases was similar in both groups (Group 1: 3.4 ± 2.9, Group 2: 3.4 ± 2.9, *p* = 0.938).

### 3.3. Comparison of ECW/TBW and Frailty Index Between Groups

A highly significant difference was observed in the ECW/TBW ratio (*p* < 0.001), with Group 2 exhibiting a higher mean value (49.4 ± 2.7%) compared to Group 1 (46.9 ± 2.6%). The TFI total score was also significantly higher in Group 2 (6.6 ± 2.7) compared to Group 1 (5.9 ± 2.9, *p* = 0.045). Similarly, a statistically important difference in the TFI social domain was reported (*p* = 0.023), with higher scores being observed in Group 2 (2.0 ± 0.8), than in Group 1 (1.7 ± 0.9). Conversely, no statistically significant differences were observed between the groups in the individual EQ-5D domains (mobility, self-care, usual activities, pain/discomfort, anxiety/depression) or the Total 1–4 score. Table 1 and Figure 1 provide a comparison of the ECW/TBW ratio and TFI scores between Group 1 and Group 2.

### 3.4. Correlation Between MNA Score and Body Composition

**Figure 2:**
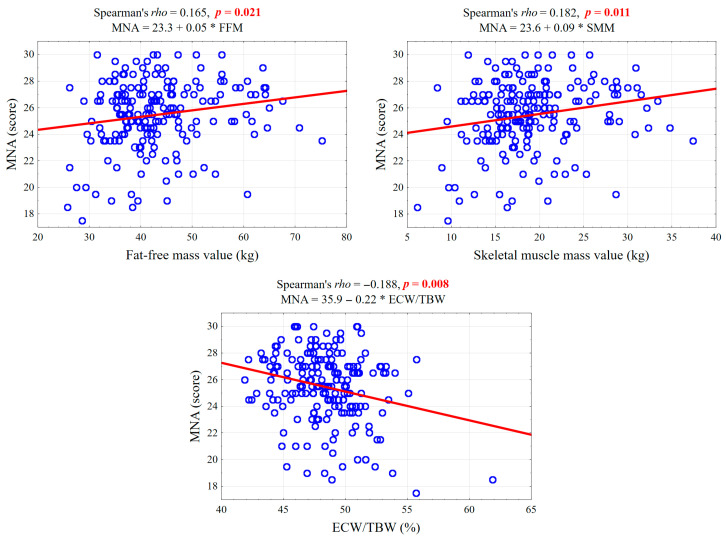
Correlation Diagrams Showing the Relationship Between Nutritional Status (MNA Score) and Body Composition Parameters, Including Spearman’s Rank Correlation Coefficients (rho) and Regression Line Equations.

A statistically significant positive correlation was found between MNA score and FFM (rho = 0.165, *p* = 0.021), indicating that higher FFM is associated with better nutritional status. Similarly, a significant positive correlation was observed between MNA score and skeletal muscle mass (rho = 0.182, *p* = 0.011), suggesting that greater muscle mass is linked to better nutrition. Conversely, there was a statistically significant negative correlation between MNA score and ECW/TBW ratio (rho = −0.188, *p* = 0.008). This implies that a higher proportion of extracellular water relative to total body water is associated with poorer nutritional status. The strongest association was observed between MNA score and ECW/TBW, as highlighted in Figure 2, where a 1% increase in ECW/TBW corresponded to an average decrease of 0.22 points in the MNA score. The correlations between MNA score and BMI (rho = 0.078, *p* = 0.280), RFM (rho = −0.026, *p* = 0.723), and AFM (rho = 0.051, *p* = 0.482) were not statistically significant. Table 2 and Figure 2 present Spearman’s rank correlation coefficients between the Mini Nutritional Assessment (MNA) score and various body composition parameters.

### 3.5. Association of Body Composition with Chronic Diseases and Lifestyle

BMI (*r*_pb_ = 0.248, *p* < 0.001), RFM (*r*_pb_ = 0.212, *p* = 0.003), and AFM (*r*_pb_ = 0.246, *p* < 0.001) showed statistically significant positive correlations with arterial hypertension. This indicates that higher adiposity is associated with a higher likelihood of this highly prevalent disease. As expected, BMI (*r*_pb_ = 0.458, *p* < 0.001), RFM (*r*_pb_ = 0.271, *p* < 0.001), AFM (*r*_pb_ = 0.391, *p* < 0.001), FFM (*r*_pb_ = 0.179, *p* = 0.012), and skeletal muscle mass (*r*_pb_ = 0.165, *p* = 0.020) all showed statistically significant positive correlations with obesity. Additionally, BMI (*r*_pb_ = 0.258, *p* < 0.001) and AFM (*r*_pb_ = 0.182, *p* = 0.011) were positively correlated with type 2 diabetes. Interestingly, FFM (*r*_pb_ = −0.196, *p* = 0.006) and skeletal muscle mass (*r*_pb_ = −0.195, *p* = 0.006) showed statistically significant negative correlations with gastroesophageal reflux disease, suggesting that lower muscle mass might be associated with a higher prevalence of this condition. A near-significant negative correlation was observed between BMI and lifestyle (rho = −0.138, *p* = 0.055), indicating a trend towards lower BMI values in individuals with healthier lifestyles. Table 3 presents the point-biserial correlation coefficients (*r*_pb_) between body composition parameters and chronic diseases (arterial hypertension, obesity, type 2 diabetes, gastroesophageal reflux disease), as well as Spearman’s rank correlation coefficient (rho) with lifestyle.

### 3.6. Predictive Ability of Body Composition for Malnutrition Risk

**Figure 3:**
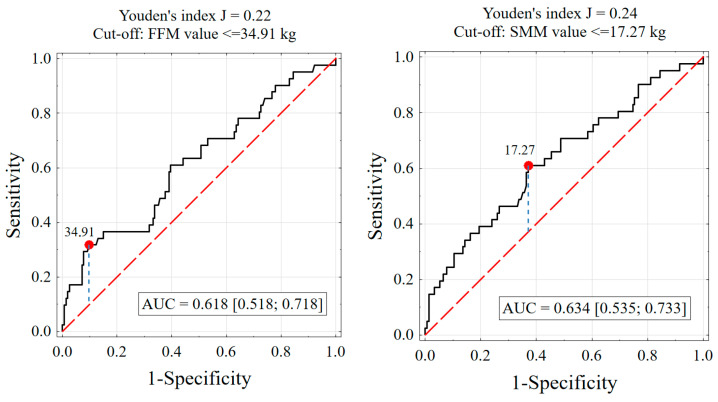
ROC Curves for Estimating the Probability of Malnutrition Risk Based on FFM Value and Skeletal Muscle Mass Value, Including the Area Under the Curve (AUC), Youden’s Index Value, and Proposed Cut-off (Threshold) Values.

In this cohort, 21.0% (n = 41) of participants were at risk of malnutrition. Skeletal muscle mass showed the highest Area Under the ROC Curve (AUC = 0.634, 95% CI [0.535; 0.733]) with a cut-off value of ≤17.3 kg, yielding a sensitivity of 0.610 and a specificity of 0.630 (LR+ = 1.65). The ECW/TBW ratio also demonstrated good predictive ability (AUC = 0.626, 95% CI [0.530; 0.722]) with a cut-off of ≥49.7%, sensitivity of 0.463, and specificity of 0.734 (LR+ = 1.74). FFM had an AUC of 0.618 (95% CI [0.518; 0.718]) and at a cut-off of ≤34.9 kg, it showed a high specificity of 0.903 but a lower sensitivity of 0.317 (LR+ = 3.26). BMI (AUC = 0.598) and AFM (AUC = 0.576) had modest discriminatory ability, while RFM (AUC = 0.513) was a poor predictor of malnutrition risk. The positive likelihood ratio of 3.26 for FFM at the identified cut-off suggests that this parameter provides fair diagnostic value in identifying individuals at risk of malnutrition. Table 4 and Figure 3 evaluate the ability of different body composition parameters to predict the risk of malnutrition, as defined by the MNA questionnaire (score ≤ 23.5).

### 3.7. Relationship Between Nutritional Status, Functionality, and Quality of Life

**Figure 4:**
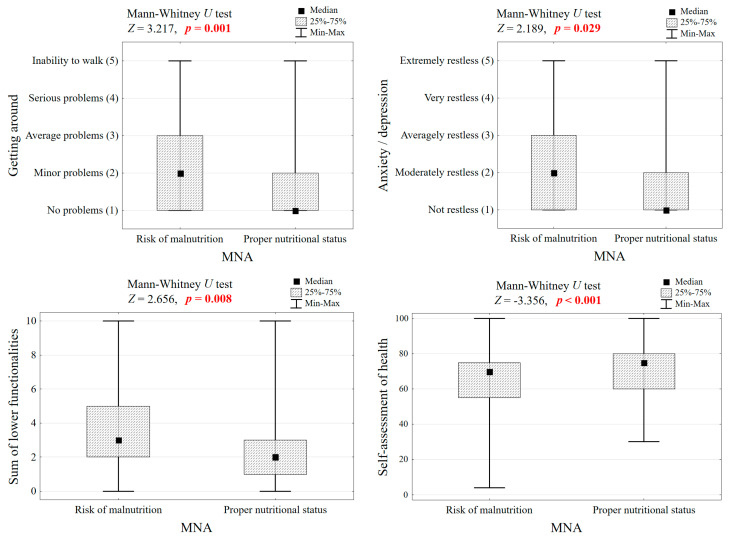
Functional limitations of seniors in groups differing in nutritional status and significance test results.

The analysis suggests that individuals at risk of malnutrition tend to experience greater functional limitations compared to those with proper nutrition, particularly in areas such as mobility and self-care. Furthermore, the figure likely demonstrates a trend towards lower quality of life scores in individuals with poorer nutritional status. While the specific statistical significance levels for these differences are not provided in the figure description, the overall trend indicates a negative association between nutritional status and both functional status as well as quality of life in this elderly population. Figure 4 illustrates the functional limitations in older adults across different nutritional status groups, along with the results of significance tests.

### 3.8. Correlation Between MNA Score and Bioelectrical Impedance Analysis Results

Several parameters showed statistically significant correlations with the MNA score (Table S1). Positive correlations were observed with SMM in the torso (r = 0.171), right leg (r = 0.221), left leg (r = 0.214), left arm (r = 0.191), and right arm (r = 0.181), as well as total body water (r = 0.202), and extracellular water (r = 0.169). Similarly, bioimpedance reactance at 5 kHz in the right leg (r = 0.175) and at 7.5 kHz in the right leg (r = 0.171), as well as bioimpedance reactance at 50 kHz in the torso (r = 0.157) were also positively correlated with MNA scores. Additionally, weight (r = 0.158), resting energy expenditure (r = 0.177), FFMI (r = 0.158), Z (FFMI) value (r = 0.195), and phase angle (r = 0.217) have all shown similar positive correlations. This may indicate that better nutritional status (higher MNA score) is generally associated with greater muscle mass, body water, reactance, weight, energy expenditure, FFMI, and a higher phase angle, which is often indicative of better cellular health and nutritional status.

## 4. Discussion

The assessment of body composition and its association with health outcomes in older adults is crucial for developing targeted interventions to promote healthy aging. This study explored how body composition, measured via bioelectrical impedance analysis (BIA), relates to nutritional status, functional limitations, and chronic diseases in older adults, comparing two groups with distinct body composition profiles.

The findings regarding nutritional status align with existing research; numerous studies confirm that MNA score shows a significant correlation with muscle mass, which was observed in our study as well [8,9]. This highlights the importance of maintaining muscle mass both as a prevention and treatment of malnutrition. It is especially important given that research indicates that the loss of muscle mass (sarcopenia) is not only common among the aging population, but is also strongly associated with functional decline, increased risk of falls, hospitalizations, and mortality [1,2]. Better acknowledgment of this relationship could lead to strengthening the preventative means and avoiding aforementioned outcomes. However, in contrast to some studies that have demonstrated a significant association between excess fat mass and poorer nutritional status, in our study the correlations between fat mass and MNA scores were weak and not statistically significant. This result may indicate that, in the geriatric population, the quality and quantity of muscle mass are more important determinants of nutritional status and functional capacity than the amount of fat tissue. This finding may be linked to the so-called ‘obesity paradox’ observed in some older populations, where a higher level of adipose tissue does not necessarily translate into worse clinical outcomes [10].

In our study, individuals with greater fat mass were more likely to suffer from metabolic diseases. This further supports the well-established role of adipose tissue—particularly visceral fat—as a key contributor to the development of metabolic syndrome [11]. On the other hand, in our study there was a lack of statistically significant associations between fat mass and certain conditions, such as type 2 diabetes, which contradicts the well-established body of research [12]. However, this discrepancy may reflect specific features of this study’s population, including variations in age, physical activity levels, disease duration, or the relatively small size of some subgroups. Findings similar to ours have been noted in other studies involving older adults, where the relationship between excess body weight and diabetes risk has not always been consistent. Differences in fat distribution with aging or compensatory mechanisms, often described as the already mentioned ‘obesity paradox’ may partly explain these inconsistencies [13].

In terms of physical functioning and quality of life, our study found that individuals with lower muscle mass and an elevated extracellular water to total body water ratio (ECW/TBW) had poorer nutritional status and greater functional limitations. The ECW/TBW ratio, also known as the edema index, is commonly considered an indirect marker of fluid retention and chronic inflammation [14,15]. Recently, it is also increasingly recognized as an indicator of muscle deterioration and frailty [4,16]. A higher value of this index may reflect a shift in fluids from the intracellular to the extracellular compartment, suggesting a decline in metabolically active tissue—both characteristic of sarcopenia and frailty syndrome [17]. Moreover, a higher ECW/TBW ratio has been associated with persistent physical discomfort, such as edema, and a decline in subjective well-being and social participation, as noted in earlier studies on older populations [18]. Lastly, our findings showed that individuals with greater fat mass tended to report lower quality of life scores (on a 0–100 scale), which is consistent with existing literature on this subject [19]. This association may stem not only from physical limitations but also from psychosocial factors, including diminished self-esteem, fear of movement, and social withdrawal.

The role of gender differences and socio-demographic factors on the nutritional status of older adults remains a subject of considerable interest. Existing literature indicates that elderly women are more likely to be affected by malnutrition compared to men [20]. This finding may be related to their longer average lifespan and, as a result, higher rates of loneliness. These associations were also confirmed in this study—the group at higher risk of malnutrition consisted predominantly of women. The same group featured elevated ECW/TBW ratio, which may indicate poorer nutritional status and the presence of chronic inflammatory processes. These differences are clinically significant, as malnutrition in older women is associated with an increased risk of physical and cognitive decline and a higher level of dependency. Contrary to some previous reports, in the present study, no significant relationship was found between educational level or net income and nutritional status. This finding may result from the relative homogeneity of the study population in terms of healthcare access, similar dietary patterns, or a limited number of individuals with extremely low socioeconomic status. It is also possible that some other factors—such as health condition, social support, or psychological factors—had a greater influence on nutritional status in this group than socio-economic variables [20,21].

This study provides a valuable contribution to the existing literature on the relationship between body composition, nutritional status, and functional capacity in the ever-growing geriatric population. One of the key innovative elements this study employed is the use of an integrated assessment combining bioelectrical impedance analysis (BIA) and the MNA screening tool. This combination allows for more accurate evaluation of nutritional status in geriatric patients. Considering the prevalence and clinical relevance of malnutrition in this group, earlier identification of malnutrition risk and more accurate classification of patients may facilitate significant change in outcomes as a result of earlier intervention. A noteworthy finding of this study is the analysis of the ECW/TBW ratio as a potential marker of declining nutritional status. The results suggest that higher values of this index are associated with lower MNA scores and greater functional limitations. As a result of this, ECW/TBW may serve as a non-invasive marker of deteriorating fluid-electrolyte homeostasis and frailty in the older adult population, and its incorporation into routine clinical practice could significantly enhance the sensitivity of diagnostic tools for identifying patients at risk of malnutrition. However, it is important to acknowledge that the ECW/TBW ratio may be influenced by factors unrelated to nutritional status, such as acute hydration changes or renal dysfunction [22]. Therefore, while ECW/TBW appears to be a promising marker, its interpretation should always be made in conjunction with clinical assessment and other diagnostic tools. The collected data offer an insight into the possible practical applications of body composition analysis—particularly using the BIA method—in assessing the clinical status of geriatric patients. At the same time, they indicate the possible directions of further research, such as deeper investigation of the role of ECW/TBW in the prevention and treatment of malnutrition and as component of comprehensive geriatric assessment.

While this study offers meaningful insights, it is important to recognize several limitations. The sample size was adequate for basic analyses; however, it restricts how broadly we can generalize the findings, particularly when considering older adults from different backgrounds or environments than those included in our research. Another important consideration is the lack of detailed data on diet and physical activity included in the analysis. We recognize that lifestyle factors play an important role in body composition and physical functioning but without direct measurement, it is impossible to fully understand their impact here. Tools like the Food Frequency Questionnaire (FFQ) or the International Physical Activity Questionnaire (IPAQ) could have added depth and clarity to the analysis. The observed weak correlation between fat mass and MNA scores also deserves attention, as it does not correspond with existing literature. This discrepancy may stem from the specific characteristics of our cohort, such as the presence of chronic diseases or variations in fat distribution, or from limitations in the methods used to assess body composition. Moving forward, studies including more diverse groups and advanced techniques like DEXA or impedance tomography would be highly beneficial. It is essential to note that, as a cross-sectional study, our work cannot determine causal relationships. The associations we observed indicate co-occurrence rather than directionality, which is a known limitation of this research design. Moreover, it is critical to note the significant demographic disparity between our clustered groups, with Group 2 being overwhelmingly female (95.7%). When analyzing our cohort’s baseline characteristics by sex, we observed no statistically significant difference in overall MNA scores between men and women. Consequently, the higher ECW/TBW ratio and elevated TFI scores observed in Group 2 are likely driven by well-documented, sex-related physiological differences rather than purely nutritional differences. Despite these limitations, the findings hold clinical value. They highlight how simple tools like BIA and the ECW/TBW ratio can be used to assess nutritional status in older adults and pave the way for more robust, prospective investigations.

## 5. Conclusions

In conclusion, our findings emphasize the importance of body composition analysis in identifying older adults at risk of malnutrition and functional decline. Tools such as bioelectrical impedance analysis (BIA) and the ECW/TBW ratio offer simple, non-invasive means to support clinical assessments. By integrating these measures into routine geriatric care, healthcare providers could intervene earlier and more effectively. While further longitudinal studies are needed to confirm these associations and refine intervention strategies, the results of this study highlight a promising direction for improving health outcomes in aging populations.

## Figures and Tables

**Table 1:** General characteristics of the studied seniors in groups with different body composition patterns.

	Total	Group 1	Group 2	Group 1 vs. 2
Age (year of life)	*N* = 195	*N* = 80	*N* = 115	
M ± SD	72.8 ± 5.4	71.8 ± 4.4	73.6 ± 6.0	
Me [Q1; Q3]	72 [69; 76]	72 [69; 74]	72 [69; 77]	*p* = 0.080
Min–Max	65–92	65–83	65–92	
Gender				*p* < 0.001
Female	157 (80.5%)	47 (58.7%)	110 (95.7%)	
Male	38 (19.5%)	33 (41.3%)	5 (4.3%)	
Education				*p* = 0.673
Basic	7 (3.6%)	4 (5.0%)	3 (2.6%)	
Secondary	98 (50.3%)	40 (50.0%)	58 (50.4%)	
Higher	90 (46.1%)	36 (45.0%)	54 (47.0%)	
Marital status				*p* = 0.006
Single	46 (23.6%)	16 (20.0%)	30 (26.1%)	
Married	74 (37.9%)	41 (51.3%)	33 (28.7%)	
Widow/widower	75 (38.5%)	23 (28.7%)	52 (45.2%)	
Housing status				*p* = 0.001
Living alone	109 (55.9%)	33 (41.3%)	76 (66.1%)	
Living with spouse	69 (35.4%)	40 (50.0%)	29 (25.2%)	
Living with family	17 (8.7%)	7 (8.7%)	10 (8.7%)	
Net income (PLN)				*p* = 0.538
Up to 1200	1 (0.5%)	0 (0.0%)	1 (0.9%)	
1201–1500	1 (0.5%)	1 (1.3%)	0 (0.0%)	
1501–1800	5 (2.6%)	1 (1.3%)	4 (3.5%)	
1801–2100	8 (4.1%)	3 (3.8%)	5 (4.3%)	
2101 and more	180 (92.3%)	75 (93.8%)	105 (91.3%)	
BMI (kg/m^2^)				*p* < 0.001
M ± SD	27.4 ± 4.3	30.4 ± 3.8	25.2 ± 3.3	
Me [Q1; Q3]	27.2 [25; 30]	29.6 [28; 33]	25.5 [23; 28]	
Min–Max	16.6–45.1	24.6–45.1	16.9–32.1	
RFM (%)				*p* = 0.948
M ± SD	39.9 ± 6.9	39.8 ± 8.0	40.0 ± 6.1	
Me [Q1; Q3]	40.8 [35; 45]	41.9 [33; 46]	40.5 [37; 44]	
Min–Max	14.2–56.7	21.8–56.7	14.2–51.0	
AFM (kg)				*p* < 0.001
M ± SD	28.9 ± 8.1	34.0 ± 8.1	25.4 ± 6.1	
Me [Q1; Q3]	28.2 [23; 34]	34.0 [28; 40]	26.0 [21; 30]	
Min–Max	9.0–54.8	17.8–54.8	9.0–37.6	
FFM (kg)				*p* < 0.001
M ± SD	43.2 ± 9.3	51.2 ± 8.1	37.5 ± 4.8	
Me [Q1; Q3]	41.3 [37; 47]	48.9 [45; 57]	37.5 [35; 40]	
Min–Max	25.8–75.2	40.8–75.2	25.8–54.1	
Skeletal muscle mass (kg)				*p* < 0.001
M ± SD	19.1 ± 5.4	23.8 ± 4.6	15.8 ± 3.0	
Me [Q1; Q3]	18.3 [16; 22]	22.8 [20; 28]	16.2 [14; 18]	
Min–Max	6.1–37.4	17.8–37.4	6.1–23.4	
MNA (score)				
M ± SD	25.5 ± 2.6	25.8 ± 2.5	25.2 ± 2.7	
Me [Q1; Q3]	25.5 [24; 27]	26.3 [25; 28]	25.5 [24; 27]	*p* = 0.081
Min–Max	17.5–30	19–30	17.5–30	
Nutritional status				*p* = 0.108
Proper nutrition	154 (79.0%)	68 (85.0%)	86 (74.8%)	
Risk of malnutrition	41 (21.0%)	12 (15.0%)	29 (25.2%)	
Self-assessment of health (score)				
*M* ± *SD*	71.9 ± 15.6	71.5 ± 15.2	72.1 ± 15.9	
*Me* [Q1; Q3]	75 [60; 80]	70 [60; 80]	75 [60; 80]	*p* = 0.767
Min–Max	4–100	30–100	4–100	
Chronic diseases in medical history:				
Arterial hypertension	91 (46.7%)	44 (55.0%)	47 (40.9%)	*p* = 0.059
Coronary heart disease	10 (5.1%)	4 (5.0%)	6 (5.2%)	*p* = 1.000
Heart failure	7 (3.6%)	1 (1.3%)	6 (5.2%)	*p* = 0.243
Atrial fibrillation/arrhythmia	23 (11.8%)	10 (12.5%)	13 (11.3%)	*p* = 0.243
Atherosclerosis	27 (13.8%)	9 (11.2%)	18 (15.6%)	*p* = 0.409
High cholesterol	72 (36.9%)	32 (40.0%)	40 (34.8%)	*p* = 0.546
Obesity	33 (16.9%)	21 (26.3%)	12 (10.4%)	*p* = 0.006
Diabetes type 1	9 (4.6%)	5 (6.3%)	4 (3.5%)	*p* = 0.491
Diabetes type 2	36 (18.5%)	19 (23.8%)	17 (14.8%)	*p* = 0.134
Thyroid diseases	48 (24.6%)	21 (26.3%)	27 (23.5%)	*p* = 0.736
Asthma	11 (5.6%)	4 (5.0%)	7 (6.1%)	*p* = 1.000
COPD	5 (2.6%)	3 (3.8%)	2 (1.7%)	*p* = 0.402
Respiratory failure	10 (5.1%)	7 (8.8%)	3 (2.6%)	*p* = 0.095
Number of chronic diseases				*p* = 0.938
*M* ± *SD*	3.4 ± 2.9	3.4 ± 2.9	3.4 ± 2.9	
*Me* [Q1; Q3]	3 [1; 5]	3 [1; 5]	3 [1; 4]	
Min–Max	0–13	0–13	0–13	
ECW/TBW (%)				
*M* ± *SD*	48.4 ± 2.9	46.9 ± 2.6	49.4 ± 2.7	
*Me* [Q1; Q3]	48.4 [47; 50]	47.0 [45; 49]	48.9 [48; 51]	*p* < 0.001
Min–Max	41.9–61.9	41.9–51.2	44.0–61.9	
TFI total (score)				
*M* ± *SD*	6.3 ± 2.8	5.9 ± 2.9	6.6 ± 2.7	
*Me* [*Q*1; *Q*3]	6 [4; 9]	5.5 [4; 8]	7 [5; 9]	*p* = 0.045
Min–Max	0–13	1–13	0–13	
TFI social domain (score)				
*M* ± *SD*	1.8 ± 0.9	1.7 ± 0.9	2.0 ± 0.8	
*Me* [*Q*1; *Q*3]	2 [1; 3]	2 [1; 2]	2 [1; 3]	*p* = 0.023
Min–Max	0–3	0–3	0–3	
Mobility	1 [1; 2]	1 [1; 2]	1 [1; 2]	*p* = 0.638
Self-Care	1 [1; 1]	1 [1; 1]	1 [1; 1]	*p* = 0.955
Usual activities	1 [1; 1]	1 [1; 1]	1 [1; 1]	*p* = 0.952
Pain/discomfort	2 [1; 3]	2 [1; 3]	2 [1; 2]	*p* = 0.111
Anxiety/depression	1 [1; 2]	1 [1; 2]	1 [1; 2]	*p* = 0.209
Total 1–4	2 [1; 3]	2 [1; 3]	2 [1; 4]	*p* = 0.305

**Table 2:** Matrix of Spearman’s rank correlation coefficients between nutritional status assessment (MNA questionnaire) and body mass components.

Correlation Between:	Spearman’s *rho*	t (N-2)	*p*
MNA (score) and BMI (kg/m^2^)	0.078	1.084	0.280
MNA (score) and RFM (%)	−0.026	−0.354	0.723
MNA (score) and AFM (kg)	0.051	0.705	0.482
MNA (score) and FFM (kg)	0.165	2.328	0.021
MNA (score) and Skeletal muscle mass (kg)	0.182	2.575	0.011
MNA (score) and ECW/TBW (%)	−0.188	−2.661	0.008

**Table 3:** Values of point-biserial correlation coefficients *r_pb_* between body composition parameters and chronic diseases.

Correlation Between:	*r*_pb_	*t* (*N*-2)	*p*
BMI (kg/m^2^) and Arterial hypertension	0.248	3.560	<0.001
RFM (%) and Arterial hypertension	0.212	3.017	0.003
AFM (kg) and Arterial hypertension	0.246	3.520	<0.001
FFM (kg) and Arterial hypertension	0.034	0.375	0.636
Skeletal muscle mass (kg) and Arterial hypertension	0.044	0.609	0.543
BMI (kg/m^2^) and Obesity	0.458	7.148	<0.001
RFM (%) and Obesity	0.271	3.917	<0.001
AFM (kg) and Obesity	0.391	5.903	<0.001
FFM (kg) and Obesity	0.179	2.532	0.012
Skeletal muscle mass (kg) and Obesity	0.165	2.329	0.020
BMI (kg/m^2^) and Type 2 diabetes	0.258	3.705	<0.001
RFM (%) and Type 2 diabetes	0.075	1.052	0.294
AFM (kg) and Type 2 diabetes	0.182	2.574	0.011
FFM(kg) and Type 2 diabetes	0.123	1.722	0.087
Skeletal muscle mass (kg) and Type 2 diabetes	0.108	1.506	0.134
BMI (kg/m^2^) and Gastroesophageal reflux disease	−0.109	−1.517	0.131
RFM (%) and Gastroesophageal reflux disease	0.040	0.559	0.576
AFM (kg) and Gastroesophageal reflux disease	−0.071	1.001	0.318
FFM(kg) and Gastroesophageal reflux disease	−1.196	−2.775	0.006
Skeletal muscle mass (kg) and Gastroesophageal reflux disease	−1.195	−2.769	0.006
BMI (kg/m^2^) and Lifestyle	−0.138	−1.934	0.055
RFM (%) and Lifestyle	−0.086	−1.119	0.232
AFM (kg) and Lifestyle	−0.113	−1.585	0.115
FFM(kg) and Lifestyle	−0.036	−0.499	0.618
Skeletal muscle mass (kg) and Lifestyle	−0.020	−0.284	0.777

**Table 4:** Comparison of body mass distribution parameters’ ability to predict malnutrition risk.

Body Composition Parameters	AUC [95% CI]	Cut-Off	Sensitivity	Specivicity	LR(+)
BMI	0.598 [0.497; 0.698]	≤23.2 kg/m^2^	0.341	0.877	2.77
Relative fat mass	0.513 [0.418; 0.607]	≤43.4%	0.756	0.364	1.19
AFM	0.576 [0.475; 0.677]	≤23.7 kg	0.415	0.766	1.77
FFM v15	0.618 [0.518; 0.718]	≤34.9 kg	0.317	0.903	3.26
Skeletal muscle mass v16	0.634 [0.535; 0.733]	≤17.3 kg	0.610	0.630	1.65
ECW/TBW	0.626 [0.530; 0.722]	≥49.7%	0.463	0.734	1.74

## Data Availability

The data presented in this study are available on request from the corresponding author due to privacy restrictions. The raw data contain sensitive patient information, however, anonymized datasets will be made available upon request.

## Supplementary Materials

The following supporting information can be downloaded at: https://www.mdpi.com/article/10.3390/nu18060969/s1, Table S1. Significant correlations between nutritional status (MNA) and selected BIA parameters.
