# Supplementary material for: Body Composition by Bioelectrical Impedance Analysis: Associations with Nutritional Status, Functional Limitations, and Chronic Diseases in Older Adults
Source: Nutrients. 2026 Mar 19;18(6):969. doi: 10.3390/nu18060969 (PMC13029748; doi:10.3390/nu18060969)
Supplement: Supplementary file 1 [file nutrients-18-00969-s001.zip › nutrients-4148796-supplementary.pdf]

Table S1. Significant correlations between nutritional status (MNA) and selected BIA parameters.

**Bioelectrical impedance results**	***r***
SMM (torso) value (kg)	0.171
SMM (RL) value (kg)	0.221
SMM (LL) value (kg)	0.214
SMM (LA) value (kg)	0.191
SMM (RA) value (kg)	0.181
Total Body Water value (L)	0.202
Extracellular water value (L)	0.169
Bioimpedance R (5 kHz) LA value (Ω)	-0.158
Bioimpedance R (5 kHz) RA value (Ω)	-0.154
Bioimpedance R (7,5 kHz) LA value (Ω)	-0.159
Bioimpedance R (7,5 kHz) RA value (Ω)	-0.156
Bioimpedance R (50 kHz) LA value (Ω)	-0.168
Bioimpedance R (50 kHz) RA value (Ω)	-0.167
Bioimpedance R (75 kHz) LA value (Ω)	-0.169
Bioimpedance R (75 kHz) RA value (Ω)	-0.170
Bioimpedance ǀXcǀ (5 kHz) RL value (Ω)	0.175
Bioimpedance ǀXcǀ (7,5 kHz) RL value (Ω)	0.171
Bioimpedance ǀXcǀ (50 kHz) TO value (Ω)	0.157
Weight value (kg)	0.158
Resting energy expenditure value (kcal)	0.177
FFMI value (-)	0.158
Z(FFMI) value	0.195
Bioelectric impedance vector analysis Z(R) value	-0.187
Phase Angle value	0.217
ECW by TBW value (%)	-0.242
